# A WHO remit to improve global standards for medical products of human origin

**DOI:** 10.2471/BLT.24.291569

**Published:** 2024-09-02

**Authors:** Eoin McGrath, Marisa R Herson, Matthew J Kuehnert, Karen Moniz, Zbigniew M Szczepiorkowski, Timothy L Pruett

**Affiliations:** a International Council for Commonality in Blood Banking Automation, Calle Balcells 29 baixos, Barcelona, 08024, Spain.; b Faculty of Health, Deakin University School of Medicine, Geelong, Australia.; c Hackensack Meridian School of Medicine, Nutley, United States of America (USA).; d International Council for Commonality in Blood Banking Automation, Redlands, USA.; e Dartmouth-Hitchcock Medical Center, Lebanon, USA.; f Department of Surgery, University of Minnesota, Minneapolis, USA.

## Abstract

In recent decades, considerable advances have been made in assuring the safety of blood transfusion and organ transplantation. However, with the increasing movement of medical products of human origin across international boundaries, there is a need to enhance global norms and governance. These products, which include blood, organs, tissues, cells, human milk and faecal microbiota, are today crucial for health care but they also pose unique risks due to their human origin, such as disease transmission and graft failure. Moreover, the demand for medical products of human origin often exceeds supply, leading to dependence on international supply chains, and emerging technologies like cell and gene therapy present further challenges because of their unproven efficacy and long-term risks. Current regulatory mechanisms, especially in low- and middle-income countries, are insufficient. The World Health Organization (WHO) has both the mandate and experience to lead the development of international quality and safety standards, consistent product nomenclature, and robust traceability and biovigilance systems. An international, multistakeholder approach is critical for addressing the complexities of how medical products of human origin are used globally and for ensuring their safety. This approach will require promoting uniform product descriptions, enhancing digital communication systems and leveraging existing resources to support countries in establishing regulations for these products. As illustrated by World Health Assembly resolution WHA77.4 on transplantation in 2024, WHO’s ongoing efforts to ensure the safe, efficient and ethical use of medical products of human origin worldwide provide the opportunity to galvanize international cooperation on establishing norms.

## Résumé

Au cours des dernières décennies, des progrès considérables ont été réalisés pour assurer la sécurité des transfusions sanguines et des transplantations d'organes. Cependant, avec l'augmentation de la circulation de produits médicaux d'origine humaine par-delà les frontières internationales, il est impératif de renforcer la gouvernance et les normes mondiales. Ces produits, parmi lesquels figurent le sang, les organes, les tissus, les cellules, le lait maternel et le microbiote fécal, sont aujourd'hui essentiels pour les soins de santé. Mais ils comportent également des risques particuliers en raison de leur origine humaine, comme la transmission de maladies et le rejet de greffe. En outre, la demande en produits médicaux d'origine humaine dépasse souvent l'offre, ce qui engendre une dépendance vis-à-vis des chaînes d'approvisionnement internationales, tandis que des technologies émergentes telles que la thérapie cellulaire et génique posent de nouveaux défis en raison de leur efficacité non démontrée et des risques à long terme. Les mécanismes de réglementation actuels sont insuffisants, surtout dans les pays à revenu faible et intermédiaire. L'Organisation mondiale de la Santé (OMS) possède à la fois le mandat et l'expérience nécessaires pour mener le développement de normes internationales de qualité et de sécurité, d'une nomenclature cohérente des produits, ainsi que de systèmes de traçabilité et de biovigilance solides. Une approche internationale et multilatérale est cruciale pour gérer la complexité liée à l'utilisation de produits médicaux d'origine humaine dans le monde et garantir leur innocuité. Cette approche devra prévoir la mise en place de descriptions de produits uniformes, l'amélioration des systèmes de communication numériques et l'exploitation des ressources existantes afin d'aider les pays à définir des règles pour de tels produits. Comme l'illustre la résolution WHA77.4 de l'Assemblée mondiale de la Santé sur la transplantation, émise en 2024, les efforts constants de l'OMS visant à assurer la sécurité, l'efficacité et l'usage éthique des produits médicaux d'origine humaine à travers le monde représente l'occasion de stimuler la coopération internationale en matière d'établissement des normes.

## Resumen

En las últimas décadas, se han realizado avances considerables para garantizar la seguridad de las transfusiones de sangre y los trasplantes de órganos. Sin embargo, con el creciente movimiento de productos médicos de origen humano a través de las fronteras internacionales, es necesario reforzar las normas y la gobernanza mundiales. Estos productos, que incluyen sangre, órganos, tejidos, células, leche humana y microbiota fecal, son hoy cruciales para la asistencia sanitaria, pero también plantean riesgos únicos por su origen humano, como la transmisión de enfermedades y el fracaso de los injertos. Además, la demanda de productos médicos de origen humano suele ser superior a la oferta, lo que hace depender de las cadenas de suministro internacionales, y las tecnologías emergentes, como la terapia celular y genética, plantean nuevos desafíos debido a su eficacia no demostrada y a sus riesgos a largo plazo. Los mecanismos reguladores actuales, en especial en los países de ingresos bajos y medios, son insuficientes. La Organización Mundial de la Salud (OMS) tiene tanto el mandato como la experiencia para liderar el desarrollo de estándares internacionales de calidad y seguridad, nomenclatura coherente de productos y sistemas sólidos de trazabilidad y biovigilancia. Para responder a la complejidad del uso global de los productos médicos de origen humano y garantizar su seguridad, es fundamental un enfoque internacional que incluya a todas las partes interesadas. Este enfoque requerirá promover descripciones uniformes de los productos, reforzar los sistemas de comunicación digital y aprovechar los recursos existentes con el fin de ayudar a los países a establecer normativas para estos productos. Como se ilustra en la resolución WHA77.4 de la Asamblea Mundial de la Salud sobre trasplantes en 2024, los esfuerzos en curso de la OMS para asegurar el uso seguro, eficiente y ético de los productos médicos de origen humano en todo el mundo brindan la oportunidad de impulsar la cooperación internacional en el establecimiento de normas.

## ملخص

في العقود الأخيرة، تم تحقيق تقدم كبير في سبيل ضمان سلامة عمليات نقل الدم وزرع الأعضاء. إلا أنه مع تزايد حركة المنتجات الطبية ذات الأصل البشري عبر الحدود الدولية، فإن هناك حاجة إلى تعزيز المعايير والحوكمة العالمية. هذه المنتجات، والتي تشمل الدم، والأعضاء، والأنسجة، والخلايا، وحليب الأم، وميكروبات البراز، أصبحت اليوم حاسمة للرعاية الصحية ولكنها تمثل أيضًا مخاطر فريدة من نوعها بسبب أصلها البشري، مثل انتقال الأمراض وفشل الزرع. وعلاوة على ذلك، فإن الطلب على المنتجات الطبية ذات الأصل البشري غالبًا ما يتجاوز العرض، مما يؤدي إلى الاعتماد على سلاسل التوريد الدولية، وتمثل التقنيات الناشئة، مثل العلاج الخلوي والعلاج الجيني، مزيدًا من التحديات بسبب فعاليتها غير المثبتة، ومخاطرها طويلة الأجل. إن الآليات التنظيمية الحالية غير كافية، وخاصة في الدول ذات الدخل المنخفض والدخل المتوسط. تتمتع منظمة الصحة العالمية (ًWHO) بالاختصاص والخبرة لقيادة تطوير المعايير القياسية للجودة والسلامة الدولية، وتسمية المنتجات بشكل متسق، وأنظمة التتبع، واليقظة البيولوجية الفعالة. إن اتباع أسلوب دولي لأصحاب المصلحة المتعددين أمر بالغ الأهمية لمعالجة التعقيدات المحيطة في كيفية استخدام المنتجات الطبية ذات الأصل البشري على مستوى العالم، ولضمان سلامتها. وسوف يتطلب هذا الأسلوب الترويج لأوصاف المنتجات الموحدة، وتعزيز أنظمة الاتصالات الرقمية، والاستفادة من الموارد الموجودة لدعم الدول في وضع اللوائح الخاصة بهذه المنتجات. وكما يتضح من قرار جمعية الصحة العالمية رقم WHA77.4 بشأن زراعة الأعضاء في عام 2024، فإن الجهود الجارية من طرف منظمة الصحة العالمية لضمان الاستخدام الآمن والفعال والأخلاقي للمنتجات الطبية ذات الأصل البشري في جميع أنحاء العالم، إنما توفر فرصة للتأكيد على أهمية التعاون الدولي من أجل وضع المعايير.

## 摘要

近几十年来，我们在确保输血和器官移植的安全方面已取得了长足进步。然而，随着越来越多的人源医疗产品跨国传输，很有必要提高全球标准要求和加强全球治理。包括血液、器官、组织、细胞、母乳和粪便微生物群在内的这类产品对现今的医疗保健行业至关重要，但是由于这类产品的人源性，也具有独特的风险，例如疾病传播和移植失败。此外，人源医疗产品往往供不应求，导致需要依赖国际供应链，同时，细胞和基因疗法等新兴技术因其疗效尚未被证实且存在长期风险而面临更大的挑战。目前的监管机制还不够完善，特别是在中低收入国家。世界卫生组织 (WHO) 需利用其以往经验，继续肩负起领导制定国际质量安全标准、确保产品命名法保持统一以及构建稳健的可追溯性和生物警戒系统的使命。需要研究出一种允许多方利益相关者参与的国际通用方法，这对于解决如何在全球范围内使用人源医疗产品这一难题以及确保其安全性至关重要。该方法要求强制采用统一的产品描述法，优化数字通信系统，并利用现有资源支持各国制定与这类产品有关的法规。如 2024 年世界卫生大会达成的第 WHA77.4 号移植相关决议所示，WHO 为确保在全球范围内安全、高效和合乎道德地使用人源医疗产品所做的持续努力，为在制定标准方面推动国际合作提供了机会。

## Резюме

За последние десятилетия были достигнуты значительные успехи в обеспечении безопасности переливания крови и трансплантации органов. Однако в связи с растущим перемещением медицинских продуктов человеческого происхождения через международные границы возникает необходимость в укреплении глобальных норм и управления этим процессом. Эти продукты, включающие кровь, органы, ткани, клетки, человеческое молоко и фекальную микробиоту, сегодня крайне важны для здравоохранения, однако в силу своего человеческого происхождения они также представляют собой уникальные риски, такие как передача заболеваний и отторжение трансплантата. Кроме того, спрос на медицинские продукты человеческого происхождения часто превышает предложение, что приводит к зависимости от международных цепочек поставок, а новые технологии, такие как клеточная и генная терапия, создают дополнительные проблемы из-за их недоказанной эффективности и долгосрочных рисков. Существующих механизмов регулирования, особенно в странах с низким и средним уровнем дохода, недостаточно. Всемирная организация здравоохранения (ВОЗ) обладает мандатом и опытом для руководства разработкой международных стандартов качества и безопасности, согласованной номенклатуры продукции, надежных систем отслеживания и биоконтроля. Международный подход с участием многих заинтересованных сторон имеет решающее значение для решения сложных проблем, связанных с использованием медицинских продуктов человеческого происхождения во всем мире, и для обеспечения их безопасности. Такой подход потребует продвижения единых описаний продуктов, совершенствования систем цифровой связи и использования существующих ресурсов для поддержки стран в разработке правил для этих продуктов. Как показала резолюция WHA77.4 Всемирной ассамблеи здравоохранения о трансплантации в 2024 году, усилия ВОЗ по обеспечению безопасного, эффективного и этичного использования медицинских продуктов человеческого происхождения во всем мире дают возможность активизировать международное сотрудничество по установлению норм.

## Introduction

In recent decades, substantial progress has been made in assuring the safety of blood transfusion and the transplantation of medical products of human origin, which are also referred to as human cells, tissues and cellular and tissue-based products or substances of human origin. However, the increasing international movement of these products, and tragic events such as the inadvertent transmission of tuberculosis to bone allograft recipients that resulted in at least 10 deaths, highlight the urgent need for further work on global norms, governance and vigilance.^1^^,^^2^ Medical products of human origin encompass all biological materials derived wholly or in part from the human body, including, but not limited to, blood, organs, tissues such as corneas and reproductive tissues, cells, human milk and faecal microbiota (Box 1). These products have saved and improved lives through the repair or replacement of biological structures or cells. The component materials are retrieved from living or deceased donors and have become integral to modern health care.^4^ Medical products of human origin differ from other medical products or devices because of their human origin and because they retain the biological characteristics of the donor, such as particular tissue constituents or cell functions or the presence of normal or pathological microbes. As a consequence, they have an intrinsic variability that gives rise to distinct quality and safety risks (Box 2). As well as graft failure, there is a risk of disease transmission to the recipient despite measures such as donor screening and in-process testing.

Box 1 **Taxonomy of medical products of human origin, 2020**

Organ: liver, heart, kidney, lung, pancreas, small bowel, organ combinations (i.e. heart–lung, kidney–pancreas, liver–kidney, liver–small bowel or multivisceral) and composite tissue grafts (i.e. face, hand or uterus)

Tissues: musculoskeletal (i.e. bone, cartilage, meniscus, osteochondral tissue, tendon or ligament), cardiovascular (i.e. blood vessels, conduits, heart valves or pericardium), ocular (conjunctiva, cornea, limbal tissue or sclera), adipose tissue, amniotic membrane, other fetal membranes, dura mater, larynx, nerve, parathyroid glands, placenta, skin, trachea and umbilical cord tissue

Cells: adipocytes, chondrocytes, dendritic cells, fibroblasts, genetically modified cells, hepatocytes, haematopoietic progenitor cells (i.e. marrow, cells collected by apheresis, cord blood or type not specified), keratinocytes, leukocytes, limbal cells, mesenchymal stem cells, olfactory mucosal cells, pancreatic islets and T-lymphocytes

Blood: whole blood, red blood cells, platelets, plasma, cryoprecipitate and granulocytes

Reproductive cells and tissues: embryos, oocytes, ovarian tissue, testicular tissue and sperm

Other products of human origin: milk, faecal microbiota and topical products of human origin

Medicinal products derived from medical products of human origin: plasma derivatives, cell-derived medicinal products, tissue-derived medicinal products and tissue- and cell-derived medicinal products

Source: Adapted from Italian National Transplant Centre and World Health Organization.^3^

Box 2 **Types of harm to recipients of medical products of human origin, 2018**

**Infections**

Viruses; bacteria (including mycobacteria); parasites; prions; and emerging pathogens

**Non-infectious blood transfusion reactions**

Acute reactions: transfusion-related acute lung injury; transfusion-associated circulatory overload; allergic transfusion reactions; acute febrile nonhaemolytic transfusion reactions; and acute haemolytic transfusion reactions

Delayed reactions: delayed haemolytic transfusion reactions; and delayed serological transfusion reactions

**Malignancy**

Carcinoma of unknown primary site; and cancer, multiple types: adrenal; blood and lymphoid; bone and cartilage; breast; cardiovascular; central nervous system; gastrointestinal; germ cell, sex cord and related tumours; head and neck; kidney and urinary track; liver, gallbladder and bile ducts; lung and lower respiratory system; neuroendocrine tumour; ovaries and fallopian tubes; pancreas; parathyroid; pleura and peritoneum; prostate; skin; soft tissue/sarcoma; thyroid; uterus, cervix and vagina

**Genetic transmission**

Via cells: cyclic neutropenia; Gaucher’ s disease; hyperthyroidism and autoimmune thyroiditis and thyrotoxicosis; alopecia areata; type-1 diabetes; atopy; autoimmune thrombocytopenia; myasthenia gravis; vitiligo; asthma; and anti-C1q antibodies of systemic lupus erythematosus

Via gametes and embryos: severe congenital neutropenia; hypertrophic cardiomyopathy; autosomal dominant cerebellar ataxia; Opitz syndrome; neurofibromatosis type 1; autosomal recessive polycystic kidney disease; congenital adrenal hyperplasia; and phenylketonuria

Source: Adapted from Italian National Transplant Centre and World Health Organization.^5^

Demand for almost all medical products of human origin outstrips supply, and almost all health-care systems face the challenge of attracting new donors, retaining existing donors and making deceased donation a more common choice. As a result, many end-users of these products rely on international supply chains. Data from the World Marrow Donor Association, for example, show that in 2022 almost half of worldwide haematopoietic progenitor cell products moved between countries.^6^ In 2023, the European Union parliament learned that an estimated 40% of plasma used in Member Countries comes from outside the European Union zone.^7^ The Eye Bank Association of America, which represents mostly tissue banks based in the United States of America, reported that their members exported 27 187 tissue products in 2023, an increase of 22% (22 238 tissues) from the previous year.^8^ Although this international cross-border movement has today become essential for treating patients around the world, health workers and regulators are still not working with a globally uniform terminology for medical products of human origin or with interoperable traceability mechanisms, both of which could weaken biovigilance.^9^

Furthermore, whereas transplantation and transfusion benefit from long-established scientific knowledge and involve lengthy clinical follow-up, emerging technologies, such as cell and gene therapy and xenotransplantation, present new challenges. For example, many novel therapies, including stem cell-based therapies, have yet to demonstrate their efficacy and, therefore, are subject to only minimal quality and safety standards. In addition, regulated clinical trials are still required.^10^ Moreover, there are product-specific quality and safety issues, such as emerging contaminants,^11^ and doubts about the short-term efficacy of treatment and the long-term risks for patients. The efficacy of medical products of human origin may be negatively affected by novel processing methods that alter the properties of biological tissues and cells, and medium- to long-term risks, such as those linked to late cell tumourigenicity, remain unclear, even for approved therapies.^12^

Xenotransplantation, whereby organs from animals are transplanted into humans, is presented as a possible solution to the shortage of medical products of human origin, but much still needs to be done before xenotransplantation can be considered a therapeutic practice rather than an experimental procedure.^13^

The risks inherent to medical products of human origin, their international movement and the innovations occurring in this field demand coherent international quality and safety standards and utilization norms. Furthermore, their safe use requires: (i) a consistent product nomenclature; (ii) traceability from donor to recipient; (iii) the effective and reproducible electronic transmission of critical information; and (iv) dependable biovigilance systems that monitor treatment outcomes and facilitate prompt intervention when an adverse event is detected.^14^

## Gaps in existing regulations

Global quality and safety standards, monitoring treatment outcomes and governance are critical for medicines and medical devices. However, important gaps remain in overseeing the collection, processing and utilization of medical products of human origin. In regulatory terms, these products present particular challenges because of their extensive movement across borders and between health-care systems, with each user or location potentially having their own nomenclature and definitions.^15^

Whereas many countries (e.g. Australia, Brazil and the United States) have successfully implemented robust national governance and quality and safety standards for blood, organs, tissues and cells,^16^^–^^18^ numerous others are still lagging, particularly low- and middle-income countries. For instance, in 2018, 73% (125/171) of reporting World Health Organization (WHO) Member States had a national blood policy,^19^ which was up from 68% (122/180) in 2016.^20^ In 2018, 66% (113/171) of reporting countries had specific legislation covering the safety and quality of blood transfusion, including 79% of high-income countries versus 39% of low-income countries.^21^ In these countries, a legal framework for the donation and use of medical products of human origin may not exist or, where legislation does exist, there may be little or no implementation.^22^^,^^23^ In high-income countries, regulations were shown to be inadequate by the contaminated blood scandals of the 1980s and 1990s. The lessons learnt from these cases subsequently led to substantial safety improvements to blood supplies in Japan, European countries and the United States of America, but there remains a concern that low- and middle-income countries are not yet equipped to ensure the same level of safe practice.^24^^,^^25^ An international approach is necessary to ensure that lessons continue to be learnt from experience and disseminated to the global community.^26^ Furthermore, although national oversight may be sufficient for medical products of human origin that do not move far from their collection point, as soon as products are exchanged between facilities, particularly facilities in different jurisdictions, differences in product terminology and traceability mechanisms may be encountered. 

Global distribution networks for human plasma, bone marrow and stem cells, corneas and other human tissues are already well established. Although these networks largely work well, biovigilance, which entails “systematic monitoring of serious adverse reactions and incidents in the transplantation chain,”^27^ may not be adequate. For medical products of human origin, biovigilance involves: (i) promptly recognizing potential disease transmission or graft failure; and (ii) rapidly identifying all other products derived from the same donor, donation, facility or facilities and shielding potential recipients. The second goal requires effective communication through interoperable digital systems that support the timely reporting and tracing of medical products of human origin across diverse systems when time is often of the essence. However, in 2023 only 12 WHO Member States in three of the six WHO regions were found to have developed a robust biovigilance system.^28^ This finding points to the existence of a gap in oversight. Furthermore, the cross-border movement of medical products of human origin has highlighted: (i) important differences in the denomination and classification of these products; and (ii) the minimal quality and safety standards recognized between countries. Both these factors make it more difficult to trace products, follow-up treatment outcomes and collect data in a timely manner. In addition, the increased use of these products globally requires – but currently lacks – a consistent and universally understood language for data collection and effective communication between health workers, regulatory bodies and stakeholders worldwide. Consistent and unambiguous product descriptions would enable regulators and clinical users to understand the type and characteristics of products.

Although there is a large body of international guidance on medical products of human origin, there are few multicountry regulatory frameworks. The 27 Member States of the European Union are subject to the 2024 substances of human origin regulation on minimal safety and quality standards for products such as blood, tissues, cells, human donor milk and faecal microbiota.^29^ In addition, in 2006 the six members of the Gulf Cooperation Council agreed to unify their respective national regulations on organs and tissues, although this alignment is still evolving.^30^ Collectively, however, members of these blocs will continue to rely on imports of some categories of medical products of human origin. For instance, European national regulators will have to determine whether imported plasma meets the same safety and quality requirements as plasma sourced from within the European Union. Consequently, as national and even regional regulation appears insufficient for addressing current and future challenges with medical products of human origin, an international and multistakeholder approach becomes essential.

## Role of WHO

WHO has both the mandate and experience to lead the establishment of: (i) international norms for the unambiguous identification of medical products of human origin; (ii) robust biovigilance systems compatible with global product distribution; and (iii) effective data capture.

Its constitution gives WHO the authority to adopt and approve normative instruments and to act as the “directing and coordinating authority on international health work.”^31^ Substantial normative work undertaken by WHO to help provide organized and standardized information for policy-makers resulted in, for example, the International Statistical Classification of Diseases and Related Health Problems; the International Classification of Functioning, Disability and Health; and the International Nonproprietary Name system for medicines. Regarding medical devices, WHO states that, “the absence of a standardized nomenclature (negatively affects)… patient safety, intended use, regulatory status, adverse events and availability.”^32^ The Organization is currently working on a nomenclature for medical devices in accordance with resolution WHA75.25 of the Seventy-fifth World Health Assembly.^33^ The absence of a nomenclature is as relevant for medical products of human origin as for medical devices, and WHO’s work on a medical device nomenclature provides a clear precedent for a similar approach to medical products of human origin.

At the request of its Member States, WHO has been issuing normative guidance on the use of medical products of human origin since the 1980s. The initial focus was on organ transplantation and later it was extended, to a variable degree, to other medical products of human origin, such as blood, reproductive tissues, donor human milk, tissues and cells. Guidance from WHO covers: (i) the use of common product terms and definitions to enable consistent data reporting; (ii) data on product utilization; (iii) the timely identification of adverse events; (iv) the standards needed to support regulatory and oversight structures; and (v) standards to ensure product traceability from donor to recipient.^34^^–^^43^ Furthermore, WHO has already begun to devise a basic tissue terminology and to prepare a global action framework for tissues,^44^ which will provide a roadmap for overcoming the main challenges blocking or limiting tissue donation and transplantation.

The work conducted by WHO to date and its mandate in this area were reinforced by the World Health Assembly resolution WHA77.4 on transplantation, which was approved in May 2024 at the Seventy-seventh World Health Assembly.^45^ The resolution requires WHO to devise a global strategy for increasing access to the donation and transplantation of cells, tissues and organs, which creates an excellent opportunity to bring together a diverse group of representatives of Member States and non-state organizations from around the world in a global effort to establish norms. Areas of work include: (i) ensuring the availability of reliable tools for the continuous monitoring of emerging risks associated with human-derived products; and (ii) enhancing coordination with, and between, national health authorities to implement global standards and actively collect data. Resolution WHA77.4 also calls for the promotion and maintenance of the *WHO Guiding principles on human cell, tissue and organ transplantation*,^43^ which is an example of building on an earlier initiative.

In addition, WHO is facilitating discussions aimed at achieving an international consensus on the regulation of advanced therapy medicinal products, which often use medical products of human origin as starting materials. Ultimately, this work will lead to the harmonization of, for example, definitions, terminology and standards for product coding, traceability and biovigilance.^46^

## Future challenges

Although WHO is well-suited to lead on international standards and biovigilance systems that enhance the traceability and safety of medical products of human origin, the Organization exercises its normative authority through authorized recommendations or more informal action by the Health Assembly, Executive Board and/or Secretariat.^47^ In fact, WHO relies on Member States to voluntarily adopt and implement its recommendations, and rarely exercises its constitutional authority to negotiate binding international law.^47^ The lack of an enforcement mechanism can lead to gaps in compliance that may hinder the effectiveness of global health standards.

Nevertheless, WHO’s power to drive coordinated global action is undeniable and is illustrated by data collection initiatives for medical products of human origin, such as the Global Database on Blood Safety, which was established in 1995. The existence of the database has greatly increased the availability of safe blood, thanks to policy, guidelines and advocacy informed by data.^48^ The Global Observatory on Donation and Transplantation established in 2007 is a similar project. Although it principally deals with organs, it also collects data on tissues and cells.^49^

Global inequality, political influence and the challenge of adapting to emerging advances in medicine and technology all have an impact on WHO’s mission. Moreover, the Organization’s capacity to act can be constrained by resource limitations, whether financial or human. The success of WHO’s global strategy for medical products of human origin also depends heavily on Member States assuming their responsibilities, such as providing the necessary financial and human resources, engaging in consultation and implementation processes and conducting agreed actions. Still, international harmonization is possible. The European Union’s experience over the past two decades shows that greater regulatory alignment on medical products of human origin can be achieved, and there is much to learn from its efforts to develop a consensus among Member States.^19^

Although not all the challenges discussed here will directly affect WHO, as more countries start to use medical products of human origin, there is an opportunity for countries to align their use of these products with established international good practice under the guidance of WHO. In addition, many of the data elements needed for reporting, analysis and improving communications globally have already been identified by WHO. Consequently, there is no need to start from zero. These elements should be incorporated into digital systems to support interoperability. Further initiatives should include: (i) strengthening collaboration between WHO Member States; (ii) making the best use of digital technologies and their interoperability; and (iii) incorporating into WHO guidance existing third-party standards and terminology sets for medical products of human origin, such as the Information Standard for Blood and Transplant (ISBT 128) managed by the International Council for Commonality in Blood Banking Automation, and the standardized terminology for assisted reproductive technology developed by the International Committee for Monitoring Assisted Reproductive Technology.^41^^,^^50^ By using existing resources, countries preparing to make therapeutic medical products of human origin available to their citizens can more quickly establish regulations and supporting structures (e.g. training for health workers, facilities and information systems), particularly if WHO can offer technical assistance.

## Conclusion

Medical products of human origin can be life-saving and life-changing for patients around the world. However, their use is associated with risks that require mechanisms to ensure their safety and quality. As these products become more widely used globally, international standardization becomes essential. One fundamental requirement is to establish and maintain a consensus-based nomenclature and definitions for medical products of human origin so that there is a universally understood language for data collection and for effective communication among health workers, regulatory bodies and other stakeholders worldwide, particularly for products that cross borders or move between health-care systems. Once this foundation has been laid, standardized product coding and unique identification should follow to aid global traceability, supported by the appropriate digital infrastructure.

Through its constitution, World Health Assembly resolutions and previous normative work in related fields, WHO has the authority to foster greater standardization of the terminology for medical products of human origin and to establish best practice for their clinical application. In addition, WHO can support the safe delivery of these products by developing global guidelines for their coding, collection, processing, storage and distribution, and by ensuring these guidelines reflect best practice. Moreover, WHO is well-positioned to facilitate international regulatory harmonization because of its ability to coordinate and bring together WHO Member States, international governmental organizations and non-state actors to achieve a common health goal. In addition to these essential measures, the safe and efficient delivery of medical products of human origin on a global scale also involves establishing and strengthening biovigilance. World Health Assembly resolution WHA77.4 on transplantation provides WHO with the opportunity to galvanize international cooperation and to combine the many elements identified in this article into a cohesive overarching framework for the safety, quality and accessibility of a range of medical products.
